# The Antioxidant Activity of *Thymus serpyllum* Extract Protects against the Inflammatory State and Modulates Gut Dysbiosis in Diet-Induced Obesity in Mice

**DOI:** 10.3390/antiox11061073

**Published:** 2022-05-28

**Authors:** Antonio Jesús Ruiz-Malagón, María Jesús Rodríguez-Sojo, Laura Hidalgo-García, José Alberto Molina-Tijeras, Federico García, Ivo Pischel, Miguel Romero, Juan Duarte, Patricia Diez-Echave, María Elena Rodríguez-Cabezas, Alba Rodríguez-Nogales, Julio Gálvez

**Affiliations:** 1 Department of Pharmacology, Center for Biomedical Research (CIBM), University of Granada, 18071 Granada, Spain; ajruiz@ugr.es (A.J.R.-M.); mjrodrisojo@correo.ugr.es (M.J.R.-S.); lhidgar@ugr.es (L.H.-G.); jalbertomolina@ugr.es (J.A.M.-T.); miguelr@ugr.es (M.R.); jmduarte@ugr.es (J.D.); albarn@ugr.es (A.R.-N.); jgalvez@ugr.es (J.G.); 2 Instituto de Investigación Biosanitaria de Granada (ibs.GRANADA), 18012 Granada, Spain; fegarcia@ugr.es; 3 Servicio Microbiología, Hospital Universitario Clínico San Cecilio, 18100 Granada, Spain; 4 Centre for Pharmacognosy and Phytotherapy, UCL School of Pharmacy, University of London, London WC1N 1AX, UK; i.pischel@ucl.ac.uk; 5 Centro de Investigación Biomédica en Red de Enfermedades Cardiovasculares (CIBERCV), Instituto Salud Carlos III, 28029 Madrid, Spain; 6 Centro de Investigación Biomédica en Red de Enfermedades Hepáticas y Digestivas (CIBEREHD), Instituto Salud Carlos III, 28029 Madrid, Spain

**Keywords:** *Thymus serpyllum*, high fat diet, obesity, antioxidant, anti-inflammatory, gut microbiota

## Abstract

Nowadays, there is an increasing interest in alternative therapies in the treatment of metabolic syndrome that combine efficacy and safety profiles. Therefore, this study aimed to evaluate the effect of an extract of *Thymus serpyllum*, containing rosmarinic acid, on high-fat diet (HFD)-induced obesity mice, highlighting the impact of its antioxidant activity on the inflammatory status and gut dysbiosis. The extract was administered daily (50, 100 and 150 mg/kg) in HFD-fed mice. The treatment reduced body weight gain, glucose and lipid metabolic profiles. Moreover, the extract ameliorated the inflammatory status, with the c-Jun *N*-terminal kinases (JUNK) pathway being involved, and showed a significant antioxidant effect by the reduction of radical scavenging activity and the mitigation of lipid peroxidation. Moreover, the extract was able to modulate the altered gut microbiota, restoring microbial richness and diversity, and augmenting the counts of short-chain fatty acid producing bacteria, which have been associated with the maintenance of gut permeability and weight regulation. In conclusion, the antioxidant activity of *Thymus serpyllum* extract displayed a positive impact on obesity and its metabolic alterations, also reducing systemic inflammation. These effects may be mediated by modulation of the gut microbiota.

## 1. Introduction

Metabolic syndrome is one of the main public health problems whose prevalence increases continuously [1] and is characterized by the combination of several conditions, including abdominal obesity, hypertension, dyslipidemia, impaired glucose tolerance and insulin resistance [2]. This cluster of metabolic disorders is associated with a higher risk of developing type II diabetes mellitus, cardiovascular diseases, non-alcoholic fatty liver disease (NAFLD) and even cancer [3]. Obesity is defined as abnormal or excessive fat accumulation, and it is characterized by a chronic low-grade inflammatory status, in which the immune system plays an important role [4,5].

Obesity-associated metabolic alterations involve different organs, including adipose tissue [6]. It has been proposed that white adipose tissue (WAT) is the central player in the mild inflammatory state that occurs in obese patients [7] because the accumulation of adipose mass promotes a higher production of free fatty acids, a hypoxia status and the release of pro-inflammatory mediators [7,8]. Moreover, an increase in adipocyte size (hypertrophy) takes place in obese patients, which is also associated with a higher number of adipocytes (hyperplasia) [9]. In fact, the increased size and number of adipocytes have been correlated with increased systemic insulin resistance [10] and systemic inflammation [11].

Additionally, changes in the gut microbiota composition and/or function, termed dysbiosis, have been reported to occur in obesity [12]. When the intestinal microbiota of lean and obese individuals is compared, a shift in the Firmicutes/Bacteroidetes ratio has been typically reported [13]; thus, obese individuals showed a decrease in the relative abundance of Bacteroidetes and an increase of Firmicutes in comparison with non-obese individuals [14]. It has been proposed that bacteria belonging to the phylum Firmicutes have a greater ability to extract energy from food than Bacteroidetes, favoring their absorption and, therefore, weight gain [15]. On the other hand, the abundance of Bacteroidetes in the gut is directly associated with the production of short-chain fatty acids (SCFAs), acetate and propionate and, to a lesser extent, butyrate [16]. These SCFAs are considered to have a beneficial impact on obesity due to their anti-obesogenic properties [16]. Moreover, obesity-associated dysbiosis has been associated with altered gut intestinal permeability, which facilitates the access of bacterial components, such as lipopolysaccharide (LPS), to the bloodstream. In fact, circulating levels of LPS have been demonstrated to be elevated in obese people and in experimental models of obesity in mice [17,18].

At present, the treatment of obesity has shown efficacy; however, long-term weight loss is difficult to achieve without the use of anti-obesity drugs. Unfortunately, most of them have adverse effects that can limit their use [19]. For this reason, there is a clear interest in the development of alternative and/or complementary strategies that combine efficacy and safety for preventing and/or treating obesity. Among these, plant extracts containing different active compounds, like polyphenols, can play a prominent role, given their antioxidant activity, ability to improve insulin resistance and modulate the obesity-associated inflammatory response, as well as to preserve the altered intestinal barrier function and restore gut dysbiosis [20,21,22,23,24].

*Serpylli herba* (Thyme, family Lamiaceae) is an officinal drug in the European Pharmacopeia composed of the dried flower aerial parts of wild thyme (*Thymus serpyllum*). It is an aromatic plant of Mediterranean flora (Europe and North Africa) commonly used as a spice and medicinal plant. Thyme is a good condiment for many aliments, and it has also been consumed as herbal tea since ancient times, thus indicating its safety in humans. The medicinal interest in thyme extract has been mainly focused on its essential oil obtained by steam distillation, commonly called oil of Serpolet. Moreover, this crude drug has also been commonly used as an infusion in traditional medicine to treat upper respiratory tract infections, in which the presence of phenolic derivatives seems to play a key role [25]. Additionally, thyme extract has evidenced anti-inflammatory, immunomodulatory and antioxidant properties, both in vivo and in vitro, showing a prominent role in preserving intestinal epithelial barrier function [26,27]. However, its potential benefits against obesity regarding its impact on microbiota composition and/or function have not yet been evaluated. Therefore, the aim of the present study was to evaluate the effects of an extract of *Thymus serpyllum* in a murine model of diet-induced obesity, focusing on the effects on inflammatory status and gut dysbiosis.

## 2. Materials and Methods

### 2.1. Plant Extract and Reagents

Dry powder extract from Wild Thyme (Herba Thymi serpylli Ph.Eur.) was produced under good manufacturing practice (GMP) conditions and provided by Finzelberg GmbH & Co KG (Andernach, Germany). The patented manufacturing process (EP2858655B1) is based on the complete removal of volatile oil substances, an exhaustive aqueous extraction and a gentle spray-drying process with the functional excipient Dextrin. The resulting extract is characterized by its standardization on 70% native extract (DER native 4-8:1) and 30% Dextrin. The chemical analysis revealed that it contains rosmarinic acid (1.8%, HPLC) and no essential oil < 0.1% *v*/*w* (Destillation Ph.Eur.), as well as other polyphenols, mainly Luteolin derivatives [28]. Moreover, the extract was free of common contaminants (pesticides, aflatoxins or heavy metals).

All chemicals were obtained from Sigma Chemical (Madrid, Spain), unless otherwise stated.

### 2.2. DPPH Scavenging Activity

The free radical scavenging activity (RSA) of the dilute extract of *Thymus serpyllum* was tested using a 1,1-diphenyl-2-picrylhydrazyl (DPPH) technique [29]. Briefly, the extract was dissolved in methanol to achieve a final concentration of 100 mg/mL, then, serial dilutions 1:2 were done until a concentration of 1.56 mg/mL of extract. The methanolic dilutions of the extract (10 µL) were mixed with 90 µL of phosphate buffer at pH = 7 and 200 µL of DPPH at 0.1 mM. Ascorbic acid (100–1.56 mg/mL) was assayed as a positive control. After 30 min of reaction at 25 °C and protected from the light, the scavenging activity of the compounds was measured at 515 nm in a Magellan^®^ Tecan Infinite F50 spectrophotometer. For each sample, the percentage of radical DPPH scavenging activity (% RSA) was determined using the following Equation (1):

RSA (%) = [Abs Blank − Abs Sample)/Abs Blank] × 100(1)

where Abs Blank and Abs Sample are the absorbance at 515 nm of the blank and the samples, respectively, being the absorbance of the blank the maximum absorbance, corresponding to the highest levels of DPPH radical. The half maximal inhibitory concentration (IC_50_) value, which is the concentration of the sample that can scavenge 50 % of DPPH free radical, was calculated for thyme extract and the positive control.

### 2.3. Animals, Diets and Experimental Design

Male C57BL/6J mice (7–9 weeks old) were obtained from Charles River Laboratories (Lyon, France), housed in Makrolon cages, maintained in an air-conditioned atmosphere (22 ± 1 °C, 55 ± 10% relative humidity) with a 12 h light/dark cycle, in the Laboratory Animal Facilities of the University of Granada, and provided with free access to tap water. Mice were fed with either a standard chow diet (13% calories from fat, 20% calories from protein, and 67 % calories from carbohydrates) (Global diet 2014; Harlan Laboratories, Barcelona, Spain) or a high fat diet (HFD) with 60 % of its caloric content derived from fat (Purified diet 230 HF; Scientific Animal Food & Engineering, Augy, France). Mice were randomly assigned to different groups (*n* = 10): control (standard diet (SD)), control diet treated with thyme extract (150 mg/kg), obese (HFD) and three obese groups treated with different doses of the thyme extract (50, 100 and 150 mg/kg). The administration of the extract (dissolved in 0.1 mL of sterile water) was performed daily by oral gavage from the beginning of the experiment. The control groups received daily the same volume of the vehicle used to administer the extract. The treatments were followed for 10 weeks and animal body weight as well as food and water intakes were regularly measured twice a week.

### 2.4. Thiobarbituric Acid Reactive Substance Assay

Lipid oxidation in the liver tissue was evaluated by measuring 2-thiobarbituric acid-reactive substances (TBARS) with the extraction method using thiobarbituric acid (TBA) dissolved in dimethyl sulfoxide (DMSO) and trichloroacetic acid at 10 % (*w*/*v*) in H_2_O and 1,1,3,3-tetramethoxypropane dissolved in ethanol. In this method, levels of malondialdehyde (MDA), which is a split product resulting from lipid peroxidation, were indirectly measured due to its capacity to react with TBA forming TBARS, a pink chromogen which is measured at 535 nm. TBARS levels were expressed as µM/mg protein in liver tissues. The protein content of the samples was determined using the colorimetric method of Bicinchoninic Acid Assay (BCA) using bovine serum albumin (BSA) as the standard.

### 2.5. Glucose Tolerance Test

One week before the mice were sacrificed, a glucose-tolerance test was performed on mice that were food deprived for 6 h. With this aim, mice received a 50% glucose solution in water at a dose of 2 g/kg of body weight by intraperitoneal (IP) injection and then the glucose tolerance test was carried out. Tail vein blood glucose was measured just before (time 0) IP injection of glucose and 15, 30, 60 and 120 min post injection. Blood glucose levels were measured using a handheld glucometer (Contour XT, Ascensia Diabetes Care, S.L., Barcelona, Spain).

### 2.6. Plasma Determinations

At the end of the treatment, the mice were sacrificed under isoflurane anesthesia. Blood samples were collected in tubes containing heparin, centrifuged for 20 min at 5000× *g* at 4 °C and the plasma frozen at −80 °C. Plasma glucose, insulin, LDL (low-density lipoprotein)-cholesterol and HDL (high-density lipoprotein)-cholesterol concentrations were measured by colorimetric methods using Spinreact kits (Spinreact, S.A., Girona, Spain). The Insulin Resistance Index was calculated according to the homeostatic model (HOMA-IR) using the following formula: Fasting Glucose (mM) × (Fasting Insulin (µunits/mL)/22.5).

### 2.7. Morphological Variables

Once mice were sacrificed, liver, colon, abdominal and epididymal fat, as well as brown fat deposits, were removed, cleaned and weighed. Liver and fat weight indices were calculated by dividing their weights by tibia length. Then, all tissue samples were frozen in liquid nitrogen and stored at −80 °C.

### 2.8. Histological Studies

Sections of liver and epididymal adipose tissue were fixed in 4% PFA and embedded in paraffin. Subsequently, the sections were stained with hematoxylin and eosin. Additionally, the liver sections were also fixed in 30% sucrose, embedded in Optimal Cutting Temperature (OCT) compound (Tissue-Tek^®^ O.C.T. Compound, Sakura^®^ Finetek, Torence, CA, USA) and frozen with isopentane at −40 °C. Later, 8 µm-thick sections were stained with oil red and hematoxylin. The pathohistological evaluation was evaluated by an independent pathologist. Adipocyte size was measured and analyzed using Fiji imaging software with the Adiposoft v1.16 plugin.

### 2.9. Vascular Reactivity Studies and NADPH Oxidase Activity

The isometric tension measurement was evaluated using thoracic aortic rings isolated from animals and suspended in the myograph (model 610M, Danish Myo Technology, Aarhus, Denmark) [30] with Krebs solution (mM: NaCl 118, KCl 4.75, NaHCO_3_ 25, MgSO_4_ 1.2, CaCl_2_ 2, KH_2_PO_4_ 1.2 and glucose 11) at 37 °C and gassed with 95% O_2_ and 5% CO_2_ (pH 7.4). Length-tension characteristics were obtained via the myograph software (Myodaq 2.01 (Danish Myotechnologies, Hinnerup, Denmark)), and the aortae were loaded to a tension of 5 mN. After a 90 min stabilization period, cumulative concentration-response curves to acetylcholine (10^−9^ M–10^−5^ M) were performed in intact rings that were pre-contracted with U46619 (10^−8^ M). Relaxant responses to acetylcholine were expressed as a percentage of pre-contraction.

Moreover, NADPH oxidase activity in aortic rings was determined by the lucigenin-enhanced chemiluminescence assay [31]. Aortic rings from all experimental groups were incubated for 30 min at 37 °C in HEPES-containing physiological salt solution (pH 7.4) of the following composition (in mM): NaCl 119, HEPES 20, KCl 4.6, MgSO_4_ 1, Na_2_HPO_4_ 0.15, KH_2_PO_4_ 0.4, NaHCO_3_ 1, CaCl_2_ 1.2 and glucose 5.5. Aortic production of O_2_^−^ was stimulated by the addition of NADPH (100 μM). Rings were then placed in tubes containing physiological salt solution, with or without NADPH and lucigenin was injected automatically at a final concentration of 5 μmol/L. NADPH oxidase activity was determined by measuring luminescence over 200 s in a scintillation counter (Lumat LB 9507, Berthold, Germany) in 5-s intervals and calculated by subtracting the basal values from those in the presence of NADPH. Vessels were then dried and dry weight determined. NADPH oxidase activity was expressed as relative luminescence units (RLU)/min/mg dry aortic ring.

### 2.10. Analysis of Gene Expression by RT-qPCR

Total RNA from liver, colon and epididymal fat samples was isolated by the NucleoZOL reagent (Macherey-Nagel, Düren, Germany). cDNA (20 ng) was obtained using oligo(dT) primers (Promega, Southampton, UK) (Table 1). cDNA amplification was performed on optical grade 48-well plates in an EcoTM Real time PCR system (Illumina Inc., San Diego, CA, USA) using the MasterMix qPCR SyGreen kit (PCR Biosystems Ltd., London, UK). To normalize mRNA expression, the expression of the housekeeping gene glyceraldehyde 3-phosphate dehydrogenase (*Gapdh*) was measured for comparative reference. The method used to calculate the relative gene expression was the ΔΔCt method.

### 2.11. Western Blotting Analysis

Liver and epididymal fat samples were homogenized, and protein concentrations were measured (BCA Protein Assay Kit, Pierce Biotechnology, Rockford, IL, USA). Protein homogenates were run on SDS-PAGE gel (Beyotime) and transferred onto a polyvinylidene fluoride (PVDF) membrane (Millipore). After 5% milk blocking, the membranes were incubated at 4 °C overnight in primary antibodies: AMPK (Cell Signaling #2532 at 1:1000 dilution), p-AMPK (Cell Signaling #4188 at 1:1000 dilution) and PPAR-γ (Cell Signaling #2435 at 1:1000 dilution) (Cell Signaling Technology, Danvers, MA, USA). β-actin (Santa Cruz Biotechnology, Heidelberg, Germany, sc-47778) at 1:1000 dilution served as the internal reference. After secondary antibodies (Abcam, Cambridge, UK) were incubated for 2 h at room temperature, the membranes were subjected to enhanced chemiluminescence for signal intensity quantification (Bio-Rad Laboratories, Alcobendas (Madrid), Spain). The acquired images were analyzed with ImageJ Fiji Software to determine the quantity of protein [32].

### 2.12. DNA Extraction and Sequencing Analysis

DNA from fecal contents was isolated. Then, stool DNA was amplified using primers of the bacterial 16S rRNA gene (V4–5) and analyzed using multiplexing on the Illumina MiSeq machine. The samples were then pooled to make one library to be quantified fluorometrically before sequencing. The resulting sequences were completed, quality-filtered, clustered and taxonomically assigned based on a 97% similarity level against the RDP (Ribosomal Database Project) using the QIIME software package (Version 1.9.1) (Knight Lab, San Diego, CA, USA). Sequences were selected to estimate the total bacterial diversity of the DNA samples in a comparable manner and were trimmed to remove barcodes, primers, chimaeras, plasmids, mitochondrial DNA and any non-16S bacterial reads and sequences <150 bp.

### 2.13. Quantification of the SCFA Concentrations in the Intestinal Luminal Contents

The analysis of the intestinal contents of short-chain fatty acids (SCFA) was performed by gas chromatography, as described previously [33].

### 2.14. Statistical Analysis

All results are expressed as the mean ± SEM. Differences between mean values were tested for statistical significance using a one-way analysis of variance (ANOVA) and post-hoc least significance tests. Differences between proportions were analyzed with the chi-squared test. All statistical analyses were carried out with the GraphPad 6.0 software package (GraphPad Software, Inc., La Jolla, CA, USA), with statistical significance set at *p* ≤ 0.05.

Moreover, different parameters were intercorrelated with the “R” statistical software package (version 4.0.0; https://www.r-project.org/, accessed on 1 December 2021) using the “rcorr()” function in the “Hmisc” package to compute the significance levels for Pearson’s correlations and the function “corrplot()”, in the package of the same name to create a correlogram. The correlation matrix was reordered according to the correlation coefficient using the “hclust” method.

## 3. Results and Discussion

Obesity has been recognized as playing a central role in the development of metabolic syndrome, including glucose intolerance and dyslipidemia, and cardiovascular diseases, such as hypertension, thus resulting in an increased risk of morbidity and mortality [1]. The etiology of obesity is multifactorial, involving a complex interaction among genetics and the environment; however, it is generally accepted that obesity results from the combination of a high-calorie low-fiber diet intake and reduced physical activity. Consequently, the first action should be recommendations for nonpharmacological management, including diet therapy and physical activity. These lifestyle and behavioral interventions aimed at reducing calorie intake and increasing energy expenditure have limited effectiveness. For this reason, other pharmacological strategies may be required. Unfortunately, although they can promote significant weight reduction, their chronic use can promote the onset of important side effects that limit their administration and efficacy [34]. In this sense, plant-based therapies have long been studied for their potential application in the treatment of obesity. Since the administration of thyme extract, which has previously been reported to exert antioxidant and anti-inflammatory properties [27], its use could be an interesting approach [35].

### 3.1. Thyme Extract Shows Antioxidant Effects by the Reduction of Radical Scavenging Activity In Vitro and the Mitigation of Lipid Peroxidation in HFD Fed Mice

**Figure 1:**
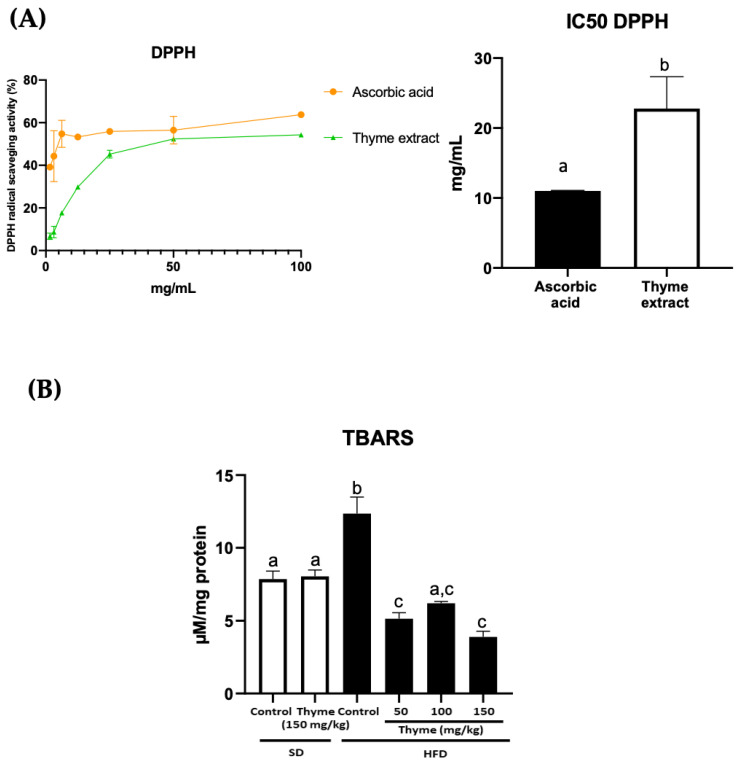
Antioxidant activity of Thyme extract: (**A**) DPPH activity scavenging of thyme extract, ascorbic acid and ferulic acid and their IC_50_ values; (**B**) TBARS production in liver lysates. Data are expressed as means ± SEM (*n* = 4). Groups with different letters statistically differ (*p* < 0.05).

The study of the in vitro antioxidant activity was carried out by analyzing the ability of the extract to neutralize DPPH, a stable organic free radical widely used for the study of the antioxidant activity of numerous compounds. The method is based on the reduction of alcoholic DPPH solution in the presence of hydrogen-donating antioxidants [36]. The thyme extract could neutralize the DPPH free radicals via hydrogen donating activity by 29.8%, 45.2%, 52.4% and 54.3%, at concentrations of 12.5, 25, 50 and 100 mg/mL, respectively (Figure 1A). The IC_50_ was found to be 22.8 ± 4.5 mg/mL for the extract, whereas the IC_50_ was 11 ± 0.1 mg/mL for ascorbic acid (Figure 1A). The results indicated that a higher percentage of DPPH scavenging activity may be attributed to the high reducing power and higher total phenolic contents present in the thyme extract.

Many studies have revealed that the consumption of an HFD causes oxidative stress in most experimental models and patients with clinical conditions [37,38]. TBARS are produced during oxidative stress, which is induced by lipid peroxidation; MDA is the best known specific TBARS and is considered a good biomarker of oxidative damage caused by free radicals in the oxidative stress process [39]. Therefore, to confirm the antioxidant effect of the thyme extract in vivo, liver samples of each mouse were taken and the levels of TBARS were evaluated. According to reported studies, our study indicated that the mice that consumed the HFD exhibited TBARS values significantly higher than both standard diet groups (Figure 1B). Conversely, the administration of thyme extract in HFD-fed mice was able to significantly reduce the TBARS values (Figure 1B). This clear decrease in lipid peroxidation restored the antioxidant status in the animals treated with thyme extract and could be attributed in part to its richness in antioxidant compounds, such as polyphenols.

### 3.2. Thyme Extract Reduces Body Weight Gain in HFD Fed Mice and Improves Glucose and Lipid Metabolic Profile

**Figure 2:**
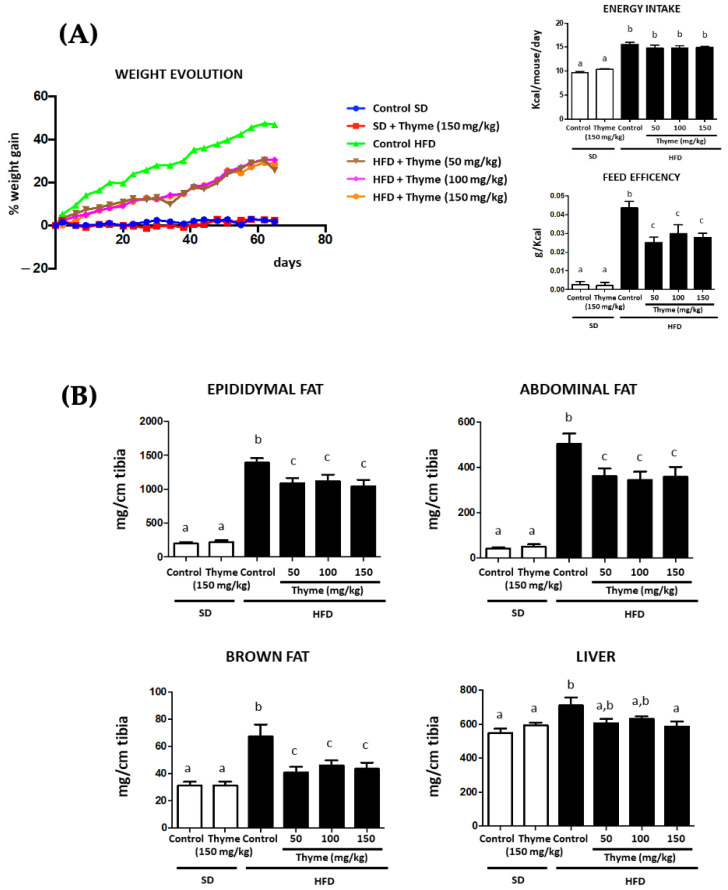
Effects of Thyme extract supplementation on: (**A**) body weight evolution, energy efficiency and energy intake and (**B**) liver and fat deposits weights. Data are expressed as means ± SEM (*n* = 10). Groups with different letters statistically differ (*p* < 0.05).

As expected, the intake of HFD resulted in a higher mouse body weight gain in the untreated control group when compared to the control group fed the standard diet (Figure 2A). The daily administration of thyme extract to HFD-fed mice significantly reduced weight gain from day 6 onwards at all doses assayed, even though food intake was similar in all HFD-fed groups throughout the experimental period (Figure 2A). Consequently, the treatments were able to significantly decrease energy efficiency in comparison with untreated HFD-fed mice (Figure 2A). Of note, the administration of the highest dose of extract (150 mg/kg) did not significantly affect the evolution of the weight of the animals that received the standard diet (Figure 2A), thus discarding any satiating effect exerted by the extract. As expected, untreated HFD mice showed heavier fat deposits (abdominal, epididymal and brown fat) than standard diet-fed mice and HFD-fed mice administered with thyme extract (Figure 2B). Interestingly, the two major types of adipose tissue, white adipose tissue (WAT) and brown adipose tissue (BAT), are functionally different. In fact, while WAT stores chemical energy in the form of fat and can secrete hormone peptides, BAT dissipates stored chemical energy in the form of heat through non-shivering thermogenesis. According to previous studies, our results indicated that long-term HFD feeding can rapidly activate BAT-mediated thermogenesis [40]. Subsequently, it has been widely reported that long-term HFD feeding in mice results in BAT dysfunction associated with tissue-specific whitening and impaired insulin sensitivity [41,42]. Moreover, strong/chronic pro-inflammatory signals can impair BAT insulin sensitivity and affect its glucose uptake, which is in turn essential for BAT function [43]. Our results are consistent with previous studies, and remarkably, treatment with the extract significantly restores the BAT increase.

In addition, liver weight was substantially increased in obese control mice compared to non-obese mice and administration of the extract at the highest dose resulted in a statistically significant reduction in liver weight (Figure 2B).

**Figure 3:**
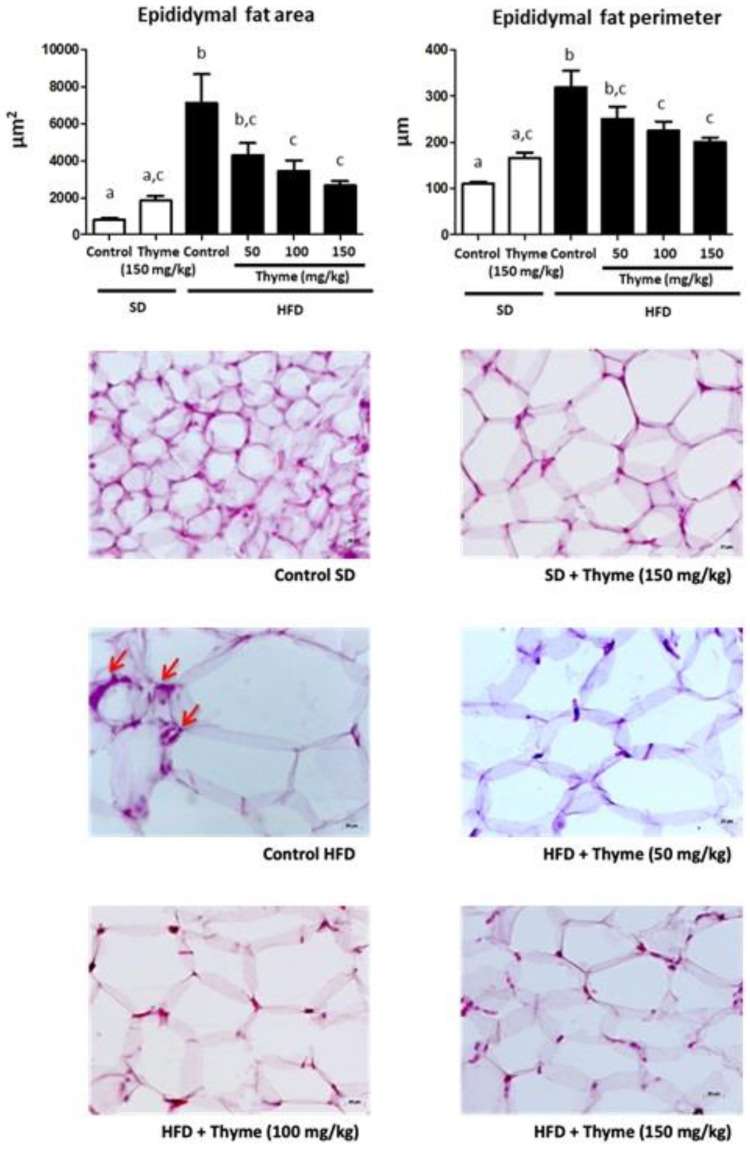
Effects of Thyme extract administration on epididymal adipose tissue, analyzed by hematoxylin and eosin staining (scale bar = 20 μm) and on area/perimeter of epididymal adipocytes. Data are expressed as means ± SEM (*n* = 10). Groups with different letters statistically differ (*p* < 0.05). Red arrows show mononuclear inflammatory aggregation.

**Figure 4:**
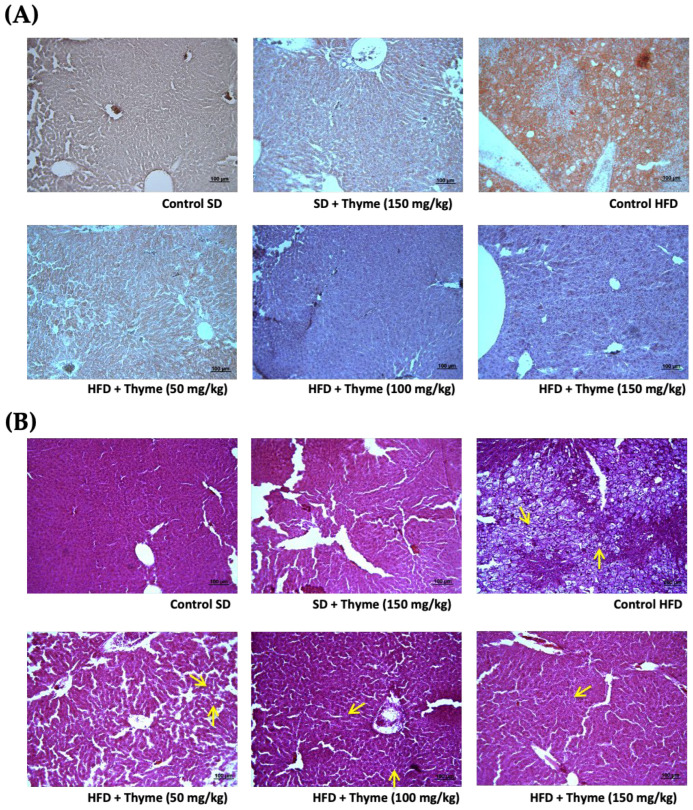
Effects of Thyme extract administration on fat deposits in liver tissue stained with oil red and hematoxylin (**A**) and with hematoxylin and eosin (**B**). Yellow arrows indicate the presence of lipid vacuoles in the cytoplasm of hepatocytes and red arrows show mononuclear inflammatory aggregation.

The histological analysis of the epididymal fat tissue from untreated HFD-fed mice showed the presence of adipocyte hypertrophy in comparison with that from standard diet-fed mice (Figure 3), which was significantly attenuated in obese mice treated with thyme extract, thus resulting in a reduction in the area and perimeter of the adipocytes (Figure 3). Similarly, the histological analysis of the liver sections revealed important obesity-associated steatosis, mainly characterized by intense fat deposition, as well as the infiltration of inflammatory cells. The oil red staining marked the lipidic droplet accumulation in the tissue, and thus, the red coloration was more evident in samples from non-treated obese mice than in non-obese mice (Figure 4A). The administration of thyme extract to HFD-fed mice clearly improved the hepatic steatosis that was evidenced in the obese mice without treatment (Figure 4A). Correspondingly, hepatic steatosis was evidenced by the increase of both fat deposition and infiltration of cells when these sections were stained with hematoxylin and eosin, since the number of fat drops observed (yellow arrows), the cell infiltration (red arrows), and the ballooning process was higher in the hepatocytes from HFD-fed mice and the administration of thyme extract resulted in a significant reduction of these steatosis markers (Figure 4B).

**Figure 5:**
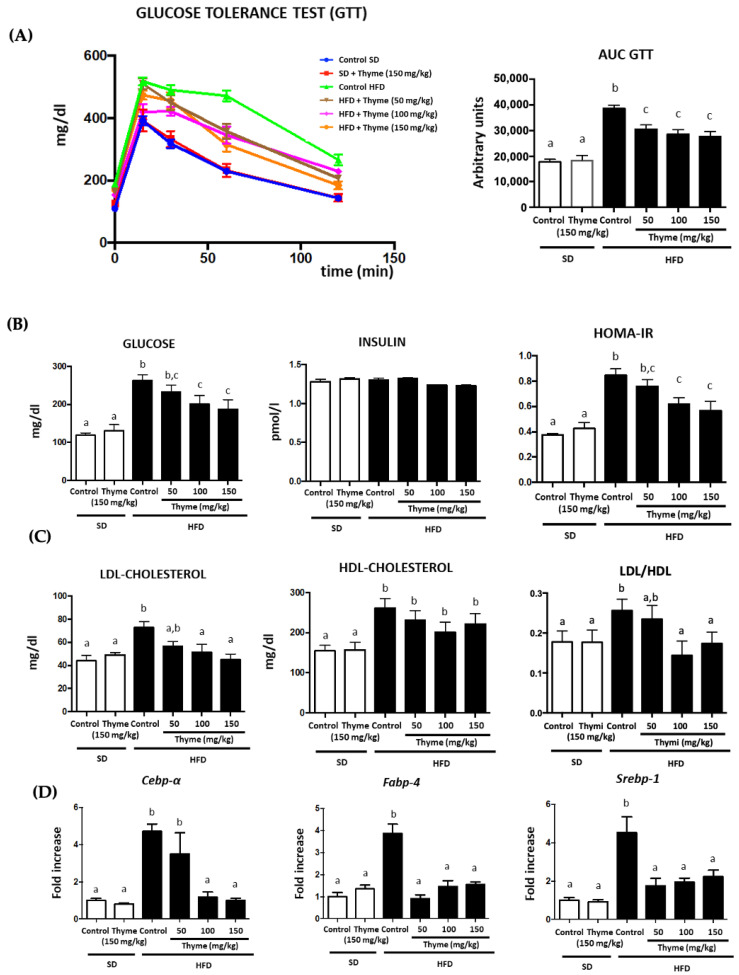
Effects of Thyme extract administration on: (**A**) glucose tolerance test (GTT), (**B**) plasma glucose and insulin levels, as well as on HOMA-IR (**C**) plasma LDL- and HDL-cholesterol levels, as well as on LDL/HDL ratio (**D**) expression of genes involved in adipogenesis in epididymal fat (*Cebp-α*, *Fabp-4* and *Srebp1*). Data are expressed as means ± SEM (*n* = 10). Groups with different letters statistically differ (*p* < 0.05).

In accordance with the macroscopic and histological results, the thyme extract also had a positive impact on the obesity-associated metabolic alterations. Glucose homeostasis was initially evaluated by the glucose tolerance test, which was performed one week before the end of the study. Control obese mice showed significantly higher glucose level peaks than non-obese groups; however, thyme extract treatment to obese mice significantly reduced plasma glucose levels in comparison with the untreated HFD group from 30 min onwards, which resulted in a significant reduction in the area under the curve (AUC) (Figure 5A). The plasma biochemical determinations performed once the mice were sacrificed confirmed the beneficial effects of thyme extract on the glycemic profile. Thus, control HFD-fed mice showed a significant increase in fasted glucose levels compared to non-obese mice, and they were significantly reduced by the administration of the extract (100 and 150 mg/kg) (Figure 5B). No significant modifications were observed when plasma insulin levels were determined in the different experimental groups (Figure 5B). However, when a homeostatic model assessment of insulin resistance (HOMA-IR) was determined, untreated obese mice showed increased values in comparison with non-obese mice, thus showing obesity-associated insulin resistance. Treatment with thyme extract (100 and 150 mg/kg) significantly ameliorated this index, which is indicative of an improvement of the insulin resistance process observed in control obese mice (Figure 5B). Similarly, control HFD-fed mice displayed modifications in lipid metabolism since obesity was associated with hypercholesterolemia in comparison with non-obese mice, and increased levels of both LDL-cholesterol and HDL-cholesterol were observed (Figure 5C). The administration of thyme extract to obese mice resulted in a significant reduction in plasma LDL levels (Figure 5C). In fact, the administration of the extract (100 and 150 mg/kg) significantly ameliorated LDL/HDL ratio in comparison with untreated obese mice (Figure 5C). In mammalian cells, the transcription factors CCAAT/enhancer binding protein α (C/EBPα), fatty acid binding protein 4 (FABP4) and sterol regulatory element-binding protein-1 (SREBP-1) are clearly involved in the regulation of adipogenesis [43,44]. The expression of these transcription factors was significantly increased in control obese mice when compared with non-obese mice, thus confirming the existence of an altered adipogenesis process in obesity (Figure 5D). Studies in vitro have previously proposed that rosmarinic acid administration suppressed the C/EBPα protein expression [45]. The administration of thyme extract resulted in an improvement in the expression of C/ebpα, Fabp4 and Srebp-1 (Figure 5D), which can be associated with the amelioration in the altered adipogenic process that occurs in obesity.

Previous studies have reported the ability of thyme to exert beneficial effects against metabolic alterations, although most of them involve the administration of its essential oil, which is attributed to the presence of phenolic compounds, such as thymol [46]. However, it is unlikely that these essential-oil derived compounds play an important role in the anti-obesogenic activity of thyme extract observed in the present study, since its content is very low (<0.01%). However, the presence of other phenolic derivatives in the extract, such as rosmarinic acid, can account for the effects described, as supported by previous studies reporting the beneficial effects shown by thyme phenolic compounds both in humans and in experimental models [47,48].

### 3.3. Thyme Extract Ameliorates Obesity-Associated Inflammation 

**Figure 6:**
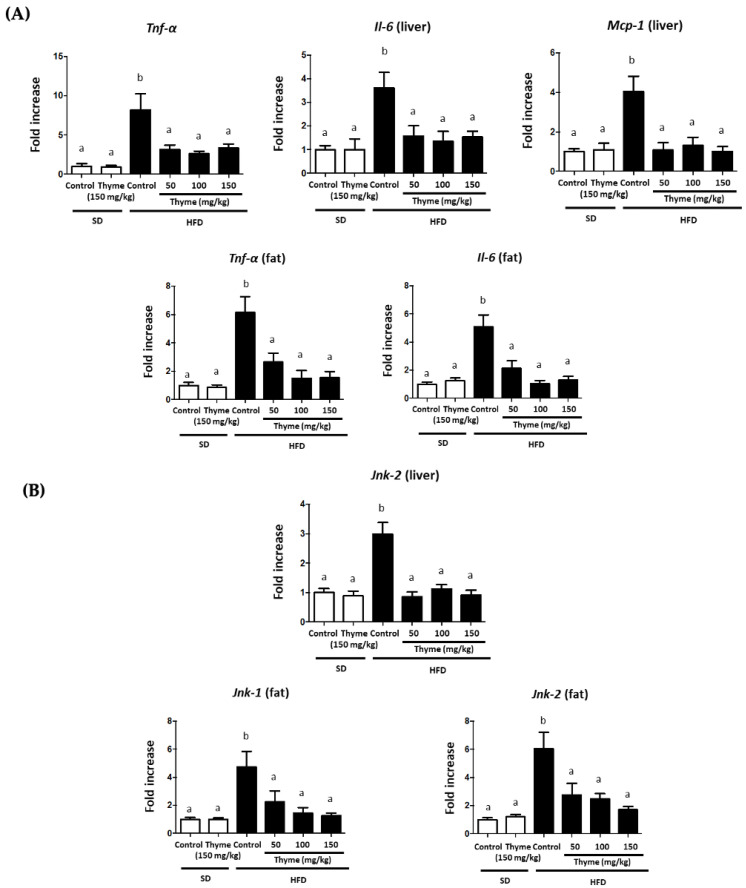
Effects of Thyme extract administration on: (**A**) gene expression of *Tnf-α* and *Il-6* in liver and fat, as well as *Mcp-1* in liver (**B**) and *Jnk-1* and *Jnk-2* in liver and fat. Data are expressed as means ± SEM (*n* = 10). Groups with different letters statistically differ (*p* < 0.05).

Different studies have reported a link between augmented body fat deposits and the development of systemic subclinical inflammation. This is associated with an increment in the levels of pro-inflammatory mediators, including cytokines, such as TNF-α or IL-6, or chemokines, such as monocyte chemotactic protein (MCP)-1, which can affect the functionality of different target tissues and organs, including liver and adipose tissue [48]. This situation promotes the stimulation of several inflammatory pathways, including those related with c-jun *N*-terminal kinases (JNKs), members of the mitogen-activated protein kinase (MAPK) that play an important role in the development of obesity-induced inflammation, impaired insulin signaling and glucose tolerance, as well as hepatic steatosis [49,50]. Furthermore, the mRNA expression of the cytokines *Tnf-α* and *Il-6* was significantly increased in liver or fat tissues from control HFD-fed mice in comparison to the non-obese control group; similarly, a significantly higher hepatic expression of the chemokine *Mcp-1* was observed in control obese mice (Figure 6A). However, the administration of thyme extract to obese mice resulted in a significant reduction in the expression of these proinflammatory markers in both the liver and fat (Figure 6A). Moreover, the expression of JNK proteins was significantly increased in control obese mice, both in the liver (*Jnk-2*) and fat (*Jnk-1* and *Jnk-2*), when compared with non-obese mice, but reduced by the administration of thyme extract (Figure 6B). Moreover, levels of PPAR-γ were studied by Western blot, and the results obtained showed that the treatment with the different doses of the extract significantly increased its levels in epididymal fat when compared with non-treated obese mice. PPAR-γ is considered one of the master regulators of adipogenesis, but it also plays an important role in controlling inflammation. In fact, PPAR-γ exerts an anti-inflammatory effect due to its capacity to interfere with proinflammatory transcription factors, including STAT, NF-κB, and AP-1, as well as with the expression of proinflammatory genes and the infiltration of macrophages. PPAR-γ is also involved in the regulation of adipogenesis, increasing the levels of adiponectin and reducing the size of adipocytes [51,52]. Consequently, the reduction in the mRNA expression of these pro-inflammatory markers, both in liver and adipose tissue, as well as the increment of PPAR-γ levels, observed after thyme extract administration of obese mice can be considered a manifestation of its anti-inflammatory properties, which could contribute to the amelioration of the altered insulin signaling and to the improvement of the glucose metabolism in obese mice. In fact, it has been reported, both in vitro and in experimental diabetes, that the ability of phenolic compounds, including rosmarinic acid, to improve obesity-related dysfunctions due to their anti-inflammatory activities through the reduction in the expression of TNFα or MCP-1 [45,48,53] and anti-adipogenic effects on adipocytes [45,53].

**Figure 7:**
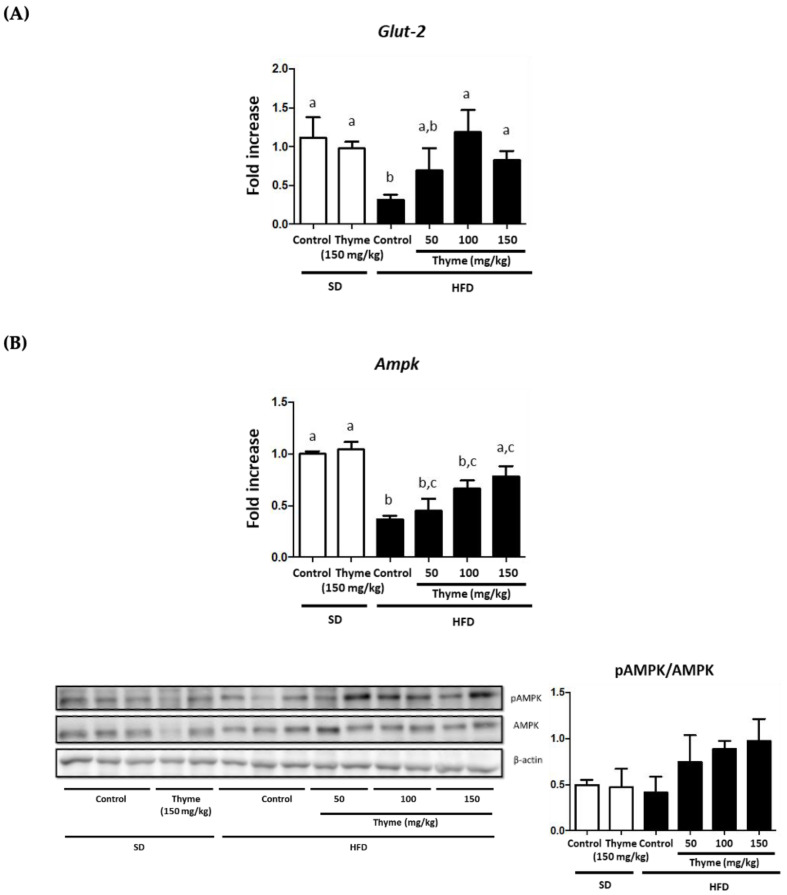
Effects of Thyme extract administration on: (**A**) gene expression of *Glut2* in liver and (**B**) *AMPK* gene expression and pAMPK/AMPK ratio evaluated by Western blot. Data are expressed as means ± SEM (*n* = 10). Groups with different letters statistically differ (*p* < 0.05).

Additionally, insulin-dependent glucose transporter type 2 (GLUT-2) is mainly expressed in the liver, thus facilitating the diffusion of glucose into hepatocytes. A reduced expression of these transporters has been reported when obesity-associated insulin resistance occurs [51], which has been confirmed in the present study. However, the administration of thyme extract significantly resulted in an increased expression of *Glut-2* in the liver (Figure 7A), thus confirming the enhancement in insulin signaling, as evidenced by the improvement of blood glucose levels in treated-obese mice. Closely related to these metabolic processes, AMP-activated protein kinase (AMPK) has been reported to have an important role in the control of inflammation and energy expenditure in obese individuals, since this enzyme facilitates catabolic pathways whereas it inhibits anabolic pathways, thus resulting in the heightening of energy expenditure [54]. Accordingly, our results support this observation, since *Ampk* gene expression was significantly reduced in the liver from untreated HFD-fed mice in comparison with those receiving a standard diet, thus revealing the situation of altered glucose and lipid metabolism in obese mice. Of note, HFD-fed mice treated with thyme extract showed an amelioration in the expression of this enzyme, although only the highest dose assayed (150 mg/kg) showed statistical differences when compared to the control obese group without treatment (Figure 7B). When the protein levels of AMPK and its phosphorylated active form (pAMPK) were evaluated by Western blot, there was an increase in the pAMPK/AMPK ratio in those obese mice treated with thyme extract (Figure 7B), thus revealing that the increased expression observed in treated obese mice was associated with higher levels of activated protein, which informs about the improvement in the altered AMPK-mediated cell signaling observed in untreated obese mice. Previous studies, performed both in experimental models of type 2 diabetes and in vitro, have reported the ability of rosmarinic acid to improve insulin sensitivity in different metabolic target organs, such as liver and skeletal muscle, via activation of AMPK, with the subsequent facilitation of the translocation of glucose transporters [55,56,57]. Consequently, the presence of polyphenolic compounds in the thyme extract, like rosmarinic acid, can clearly contribute to ameliorate glucose and lipid metabolism impairment in obesity.

**Figure 8:**
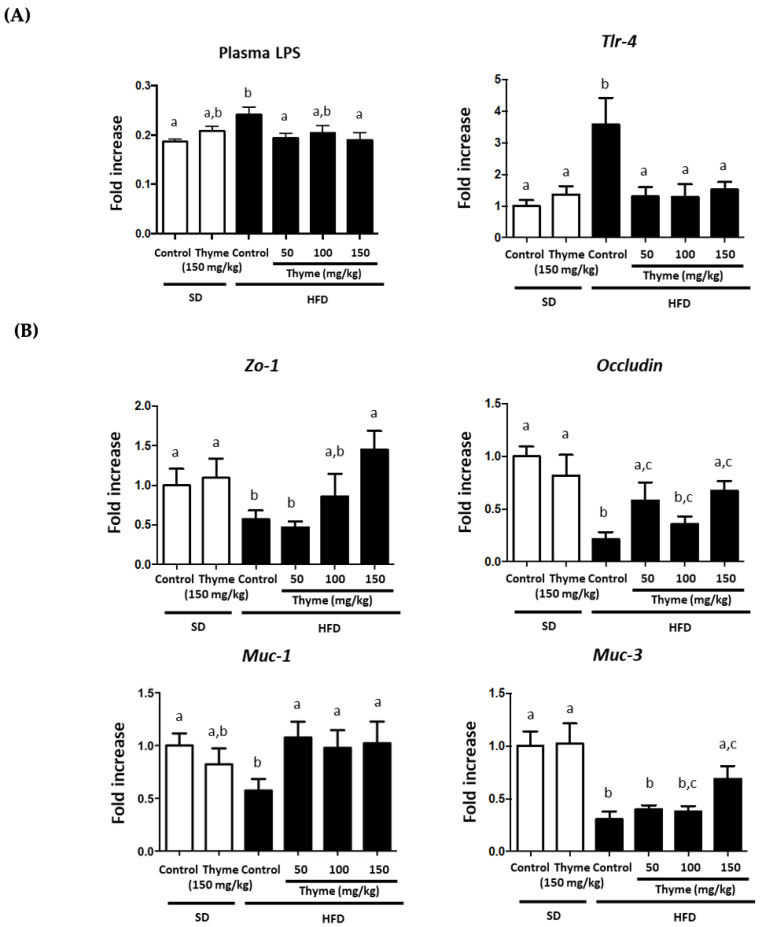
Effects of Thyme extract administration on: (**A**) plasma LPS levels and gene expression of *TLR4* in liver and (**B**) markers of intestinal barrier integrity *Zo-1*, *Occludin*, *Muc-1* and *Muc-3.* Data are expressed as means ± SEM (*n* = 10). Groups with different letters statistically differ (*p* < 0.05).

As noted above, obesity is characterized by a chronic low-grade inflammatory status, which has long been linked to increased levels of plasma bacterial LPS that promotes metabolic endotoxemia [58], and correlates with increased expression of its receptor, the toll-like receptor 4 (TLR-4) [59]. In fact, LPS activation of TLR-4 triggers different cell pathways that promote the expression and release of various pro-inflammatory cytokines [60]. The present study confirms these observations, since control obese mice showed higher plasmatic levels of LPS than non-obese mice, as well as increased expression of *Tlr-4* in the liver (Figure 8A). Of note, the administration of thyme extract to obese mice significantly reduced both plasma LPS levels and *Tlr-4* expression, thus revealing an improvement in obesity-associated metabolic endotoxemia and, consequently, in systemic inflammatory status, as evidenced by the amelioration of the expression of the different proinflammatory markers in the metabolic organs evaluated. Moreover, it has been suggested that the origin of this metabolic endotoxemia relies on an increased intestinal permeability that would promote the entry of bacterial components from the gut lumen to blood [12,61]. In fact, an alteration of the intestinal epithelial barrier function has been observed in obese mice, which downregulates the expression of different proteins associated with intestinal integrity, including *Muc-1*, *Muc-3*, *Occludin* and *Zo-1*, in comparison with non-obese mice (Figure 8B). This confirms previous studies in which obesity was associated with a reduction of the expression of tight junctions and mucin genes in intestinal epithelial cells and increased gut permeability [62]. However, the administration of thyme extract to obese mice significantly ameliorated it, thus reducing the translocation of LPS from the intestinal lumen to systemic circulation (Figure 8B). The ability of plant extracts to improve intestinal permeability has been previously described in experimental models of obesity [22,23,63] and has been attributed to the presence of phenolic compounds that have been reported to exert similar effects in experimental models of obesity [64].

### 3.4. Thyme Extract Treatment Restores Gut Dysbiosis in HFD Fed Mice

**Figure 9:**
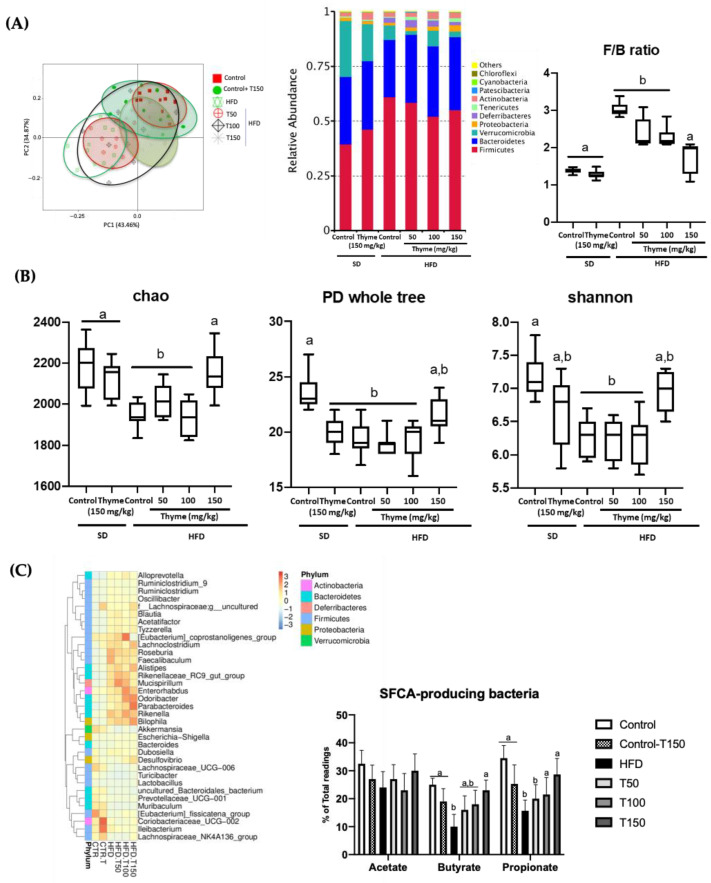
Effects of Thyme extract administration on: (**A**) Beta-diversity by principal coordinate analysis score plot, bacterial community (phyla, class and genus) and the F/B ratio; (**B**) microbiome diversity (Chao1, PD whole tree and Shannon index) and (**C**) short fatty chain acids (SCFA) production by each group of mice. Data are expressed as means ± SEM (*n* = 10). Groups with different letters statistically differ (*p* < 0.05).

It is important to note that previous studies have demonstrated the correlation among obesity, endotoxemia, increased gut permeability and gut dysbiosis [65]. In fact, obesity-associated dysbiosis was observed when a microbiome analysis of the intestinal contents was performed in the different experimental groups (Figure 9). Accordingly, the principal coordinate analysis (PCoA) plot, which represents β-diversity, revealed clear differences between control diet and untreated HFD mice diversities (Figure 9A). Of note, the PCoA plot showed a higher association among those obese mice treated with the two highest doses of thyme extract (100 and 150 mg/kg) and the control diet-fed mice than the HFD-fed mice group (Figure 9A). The most abundant phyla were Firmicutes, Bacteroidetes and Cyanobacteria, and their abundance was modified in untreated HFD-fed mice in comparison with non-obese mice, since Firmicutes was increased whereas Bacteroidetes was reduced (Figure 9A), being these changes ameliorated by the administration of the highest doses of thyme extract to obese mice (Figure 9A). The ratio of Firmicutes to Bacteroidetes (F/B), widely used as a marker of gut dysbiosis, was significantly increased in the untreated HFD-fed group compared to the standard diet-fed group, and significantly reversed in those obese mice treated with the highest doses of thyme extract (Figure 9A). Additionally, several ecological features of the gut bacterial communities were evaluated in the different experimental groups by different parameters, including Chao1 richness (diversity estimation), phylogenetic diversity (PD) whole tree (consider the phylogeny to estimate diversity across a tree), and Shannon diversity (a richness and evenness estimator). Microbial richness, evenness and diversity were significantly decreased in the HFD group compared to the standard diet group, whereas the treatment of obese mice with the highest doses of thyme extract resulted in a restoration of all these ecological parameters, obtaining similar values to those in non-obese mice (Figure 9B). At the genus level, untreated HFD-fed mice showed an increased proportion in the sequences of different genera: *Faecalibaculum* and *Roseburia* (phylum Firmicutes), *Mucispirillum* (phylum Deferribacteres) and *Rikenella* (phylum Bacteroidetes), in comparison with non-obese mice. Of note, these proportions were significantly ameliorated in obese mice treated with Thyme extract (Figure 9C). Among these genera, *Faecalibaculum* and *Roseburia* have also been reported to be increased in experimental obesity [66] being *Faecalibaculum* levels positively correlated with serum lipid levels [67]. In addition, both *Faecalibaculum* and *Roseburia* include proinflammatory bacteria that can impair gut barrier function [68]. In consequence, the restoration of these bacteria levels observed in those mice treated with thyme extract could be associated with its beneficial effects in HFD-mice, including restoration of gut barrier integrity, amelioration of the systemic inflammatory response and improvement of the lipid and glucose metabolic function. Moreover, when the proportions of SCFA-producing bacteria were evaluated, HFD-fed mice showed reduced counts of these bacteria compared with those obtained in non-obese mice (Figure 9C). The treatment with the extract resulted in an improvement in the counts of these bacteria, obtaining statistical significance in those involved in butyrate and propionate production at the highest doses assayed (150 mg/kg) (Figure 9C). It is important to note that these SCFAs regulate pH in the colonic lumen, thus clearly modulating the composition of the gut microbiota. In fact, high SCFA concentrations prevent the overgrowth of pH-sensitive pathogenic bacteria and are related to weight regulation by controlling food intake [16], enhancement of intestinal barrier integrity [69] and anti-inflammatory effects [70].

### 3.5. Thyme Extract Administration Enhances Endothelial Function

**Figure 10:**
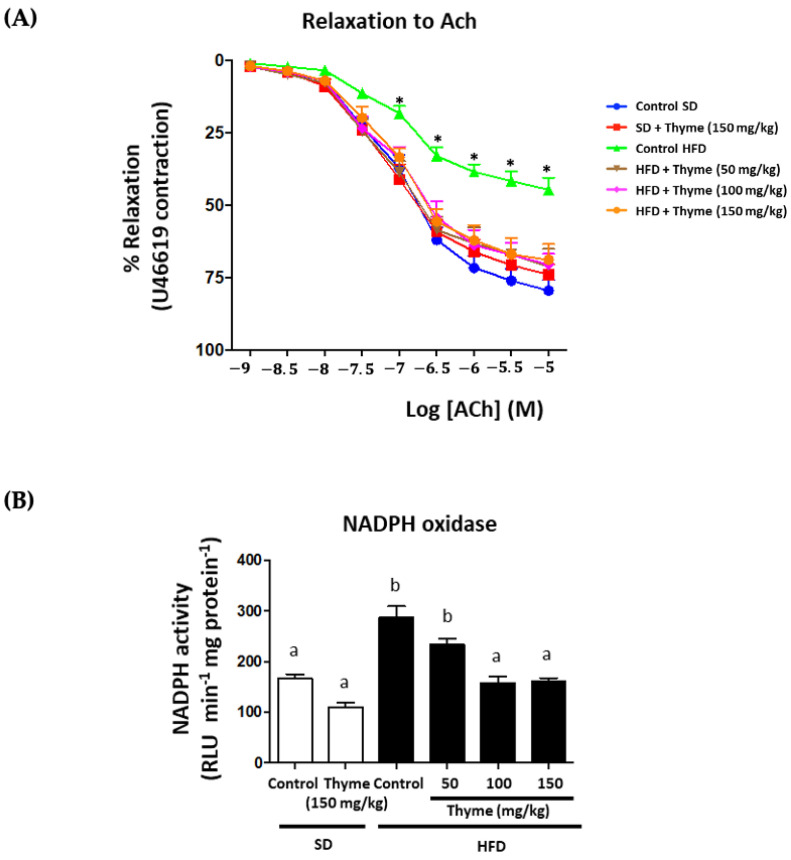
Effects of Thyme extract administration on aortic endothelial function: (**A**) endothelium-dependent relaxation to acetylcholine after contraction with U46619; (**B**) aortic NADPH oxidase activity. Data are expressed as means ± SEM (*n* = 10). Groups with different letters statistically differ (*p* < 0.05).

Finally, aorta endothelium-dependent vasodilator responses to acetylcholine were analyzed to evaluate the impact of the treatment in obesity-associated cardiovascular dysfunction. HFD-fed mice showed a reduction in the maximal relaxant response in comparison with control mice (Emax values were 44.5 ± 3.9% and 79.4 ± 3.8% in the HFD and SD groups, respectively; *p* < 0.05) (Figure 10). The administration of the extract to obese mice, at all doses, significantly improved the altered endothelium-dependent relaxation induced by acetylcholine, without obtaining statistical differences in the Emax values in comparison with those in the control diet-fed mice (Figure 10). In addition, when NADPH oxidase activity was evaluated in aortic rings, this was significantly higher in HFD mice than in non-obese mice (Figure 10); however, the treatment of obese mice with thyme extract significantly decreased it, thus improving the generation of the superoxide anion in the aortic tissue from obese mice. This effect seems to be crucial to ameliorate vascular dysfunction in obese mice because the enzyme NADPH oxidase has been proposed as the main source of reactive oxygen species in vessels. These are the most relevant products involved in the impairment of endothelium-dependent vasodilation in obesity-associated cardiovascular conditions [71]. It has been previously reported that the ability of different plant extracts to improve vascular function in experimental obesity is mainly attributed to the presence of polyphenolic derivatives [17,22,63]. In fact, among these phenolic compounds, flavonoids like quercetin or epicatechin have been described to prevent endothelial dysfunction through inhibition of NADPH oxidase activity [72,73]. Consequently, the presence of phenolic derivatives in thyme extract can account for its beneficial effects against obesity-associated vascular dysfunction.

**Figure 11:**
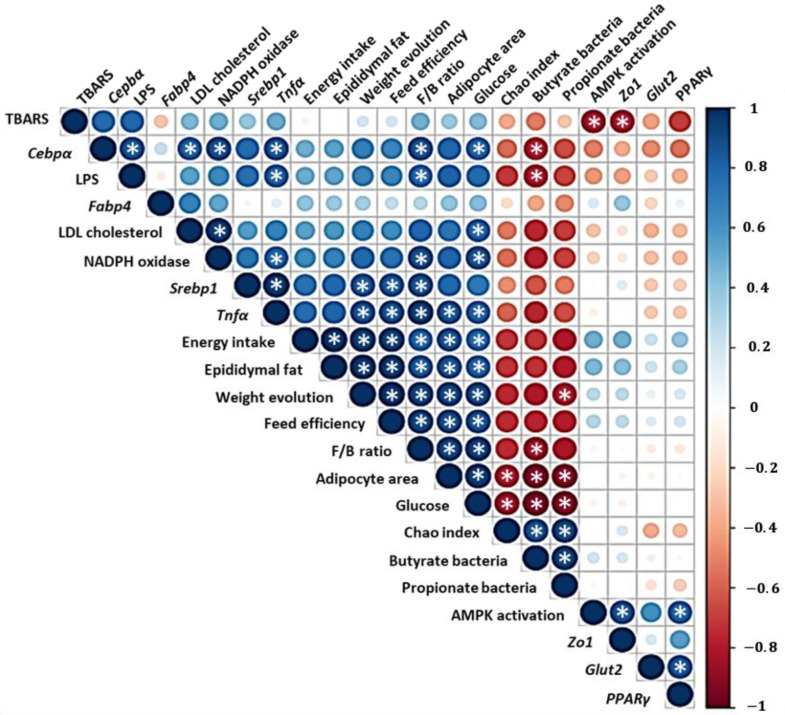
Correlation analysis was performed according to Pearson’s correlation of all data generated in the present study. Strength of the association is denoted by both the colour and the size of the bubble (darker colour and bigger size meaning higher correlation; red, negative; blue, positive). White asterisks indicate statistically significant association (* *p* < 0.05).

Lastly, the correlation between main metabolic mediators and markers of inflammation related to obesity, microbial parameters and the antioxidant activity of the extract using the non-parametric test of Spearman confirmed that beneficial effects observed thyme extract-treated mice could be derived from a synergistic effect of all the mechanisms mentioned (Figure 11).

## 4. Conclusions

In conclusion, our results indicate that *Thymus serpyllum* extract has a beneficial effect on high fat diet-induced obesity in mice, ameliorating its metabolic alterations and reducing systemic inflammation, which plays a central role in their development. These effects may be mediated by its antioxidant activity, the modulation of the gut microbiota, reducing proinflammatory bacteria and promoting those with anti-inflammatory properties and by the regulation of adipogenic-related mediators and the maintenance of gut permeability. Therefore, the results of this study are in support of further investigations into the therapeutic potential of *Thymus serpyllum* extract in metabolic syndrome treatment, as well as obesity-associated cardiovascular dysfunction and modulation of gut microbiota.

## Figures and Tables

**Table 1:** Primer sequences used in real-time PCR assays.

Gen	Sequence 5′-3′	Annealing Temperature (°C)	Accession Number
*Gapdh*	F: CCATCACCATCTTCCAGGAG R: CCTGCTTCACCACCTTCTTG	60	NM_001289726.1
*Cebp-α*	F: AAGGGTGTATGTAGTAGTGG R: AAAAAGAAGAGAAGGAAGCG	55	NM_001287523
*Fabp-4*	F: AGCATCATAACCCTAGATGG R: CCTTTCATAACACATTCCACC	55	MW286445
*Srebp-1*	F:CTGTGTGATCTACTTCTTGTG R: CATGTAGGAATACCCTCCTC	55	BC056922.1
*Tnf-α*	F: AACTAGTGGTGCCAGCCGAT R: CTTCACAGAGCAATGACTCC	60	NM_001278601.1
*Il-6*	F: TAGTCCTTCCTACCCCAATTTCC R: TTGGTCCTTAGCCACTCCTTCC	60	NM_031168.2
*Jnk-1*	F: GATTTTGGACTGGCGAGGACT R: TAGCCCATGCCGAGAATGA	60	AY383616.1
*Jnk-2*	F: TTGTGCTGCTTTTGATACAGTTCTTGGG R: CTGGAAAGAGCTCTTCAAATTTGAT	62	NM_207692.2
*Ampk*	F: GACTTCCTTCACAGCCTCATC R:CGCGCGACTATCAAAGACATACG	60	XM_036159053.1
*Glut-2*	F: TCAGAAGACAAGATCACCGGA R: GCTGGTGTGACTGTAAGTGGG	59	NM_031197.2
*Tlr-4*	F: GCCTTTCAGGGAATTAAGCTCC R: AGATCAACCGATGGACGTGTAA	60	NM_021297.3
*Muc-1*	F: GCAGTCCTCAGTGGCACCTC R: CACCGTGGGCTACTGGAGAG	60	NM_013605.2
*Muc-3*	F: CGTGGTCAACTGCGAGAATGG R: CGGCTCTATCTCTACGCTCTCC	60	NM_005960.1
*Occludin*	F: ACGGACCCTGACCACTATGA R: TCAGCAGCAGCCATGTACTC	56	U49185.1
*Zo-1*	F: GGGGCCTACACTGATCAAGA R: TGGAGATGAGGCTTCTGCTT	56	D14340.1

## Data Availability

The data presented in this study are available on request from the corresponding author. The data are not publicly available due to additional experiments are in progress to better characterize the properties of Thyme extract in metabolic syndrome.
